# Profound hyponatremia and dehydration: A case of cisplatin induced renal salt wasting syndrome

**DOI:** 10.14814/phy2.15617

**Published:** 2023-03-03

**Authors:** Tissa Bijoy George, Kiran Kuriakose, Anjana Chandrasekhara Pillai, Thomas Powell, Thomas R. Kleyman

**Affiliations:** ^1^ Department of Internal Medicine, UPMC McKeesport Pittsburgh Pennsylvania USA; ^2^ Department of Hospital Medicine, UPMC Mercy Pittsburgh Pennsylvania USA; ^3^ PINE Nephrology, UPMC McKeesport Pittsburgh Pennsylvania USA; ^4^ Department of Medicine, Department of Cell Biology, Department of Pharmacology and Chemical Biology University of Pittsburgh Pittsburgh Pennsylvania USA

**Keywords:** cisplatin, hyponatremia, renal salt wasting

## Abstract

Cisplatin is a well‐known chemotherapeutic agent that can be associated with hyponatremia. It is known to be associated with a multitude of renal disorders including acute kidney injury with reduced glomerular filtration, Fanconi syndrome, and renal tubular acidosis, nephrogenic diabetes insipidus and renal salt wasting syndrome. We report a case of an elderly male presenting with significant recurrent hyponatremia, and prerenal azotemia. With recent exposure to cisplatin along with significant hypovolemia and urinary loss of sodium, he was diagnosed to have cisplatin induced renal salt wasting syndrome.

## Graphical abstract

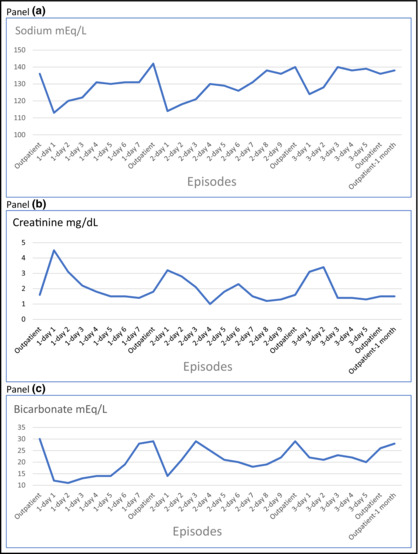

## 1 INTRODUCTION

Cis‐diamminedichloroplatinum (CDDP), commonly known as cisplatin, is a chemotherapeutic agent used in the treatment of various carcinomas, germ cell tumors, lymphomas and sarcomas. Cisplatin is known to be associated with Fanconi syndrome and renal tubular acidosis, nephrogenic diabetes insipidus, acute kidney injury with reduced glomerular filtration and renal salt wasting syndrome (RSWS) (Pham et al., 2017). In a recent literature review, Hamdi et al. analyzed 17 patients who were reported with cisplatin induced RSWS. Patients developed RSWS as soon as 12 h and up to 4 months following cisplatin therapy (Hamdi et al., 2010). We report a case of RSWS with recurrent profound hyponatremia and prerenal azotemia beginning 3 months after Cisplatin therapy.

### 1.1 Case presentation

A 63‐year‐old male patient presents to the hospital with complaints of one‐day history of dizziness and weakness. His outpatient blood work demonstrated a serum sodium level of 124 mEq/L and a creatinine of 3.1 mg/dL.

**FIGURE 1:**
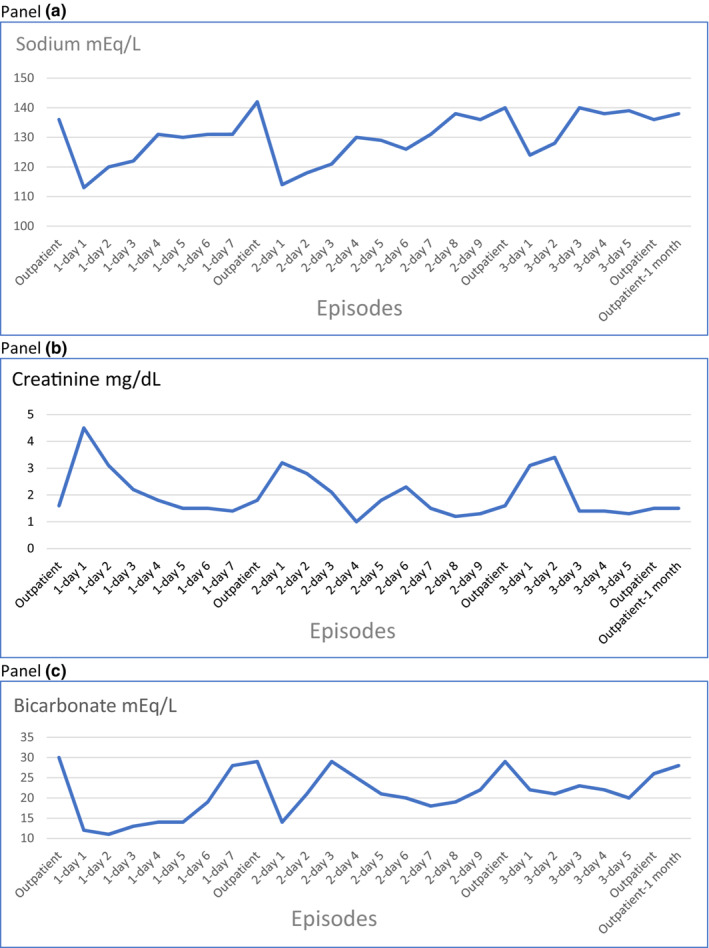
Trends in sodium, creatinine, bicarbonate levels.

His medical history was significant for high‐grade urothelial bladder carcinoma status post radical cysto‐prostatectomy, neobladder urinary diversion and MVAC (methotrexate, vinblastine, doxorubicin, and cisplatin) neoadjuvant chemotherapy for 6 months that he completed successfully in July 2020. Since October 2020, the patient was hospitalized twice for profound hyponatremia (serum sodium on presentation 113, 114 mEq/L) and prerenal azotemia (serum creatinine on presentation 4.5, 3.2 mg/dL), hyperchloremic metabolic acidosis and hyperkalemia. On both occasions, the laboratory abnormalities were reversible with intravenous isotonic fluids and he was discharged home with sodium bicarbonate tablets and a high salt diet (Figure 1). Despite adequate compliance and good oral intake, he continued to present with symptomatic hyponatremia and volume depletion. Although profoundly volume depleted on presentation, his urine sodium and osmolality were found to be elevated. He did not have glucosuria or hypophosphatemia. Of note, renal imaging and urinalysis were normal, and adrenal insufficiency was ruled out.

During the current admission, the patient presented with a serum sodium of 124 mEq/L, BUN of 143 mg/dL and creatinine of 3.1 mg/dL (Table 1 and Figure 1). This scenario of increased loss of sodium in the setting of hypovolemia suggested renal salt wasting. The possibility of Cisplatin induced renal salt wasting was considered. The patient was started on Fludrocortisone 0.1 mg twice daily, oral salt tablets 1 g QID, sodium bicarbonate tablets 650 mg TID and normal saline at 150 mL/h. During his five‐day hospital course, his renal parameters steadily improved with discharge BUN and creatinine of 60 mg/dL and 1.4 mg/dL, respectively. Likewise, his serum sodium improved from 124 to 139 mEq/L (Table 1 and Figure 1). The patient was discharged on the aforementioned medications and with recommendations to consume electrolyte‐rich fluids, a high protein and high sodium diet and obtain weekly chemistries.

**TABLE 1:** Laboratory values on admission and discharge.

	Episode 1	Episode 2	Episode 3
Admission			
Sodium	113 mEq/L	114 mEq/L	124 mEq/L
BUN	109 mg/dL	168 mg/dL	143 mg/dL
Creatinine	4.5 mg/dL	3.2 mg/dL	3.1 mg/dL
Bicarbonate	12 mEq/L	14 mEq/L	22 mEq/L
Urine osmolality		347 mOsm/kg	
Urine sodium		81 mEq/L	
Urine creatinine			
Discharge			
Sodium	131 mEq/L	136 mEq/L	139 mEq/L
BUN	46 mg/dL	47 mg/dL	49 mg/dL
Creatinine	1.4 mg/dL	1.3 mg/dL	1.3 mg/dL
Bicarbonate	28 mEq/L	22 mEq/L	20 mEq/L

Although he has had mild fluctuations in his serum chemistries based on compliance and his ability to hydrate, further hospitalizations have been avoided over an eleven‐month follow‐up. In that time frame, his serum sodium has fluctuated between 131 to 142 mEq/L and serum creatinine 1.2 to 1.8 mg/dL. Unfortunately, attempts to wean salt tablets and fludrocortisone have failed due to recurrent dizziness, hyponatremia, and rising creatinine values.

## 2 DISCUSSION

Cisplatin is a well‐known chemotherapeutic agent that can be associated with hyponatremia. Mechanisms of cisplatin‐induced hyponatremia include renal salt wasting syndrome (RSWS), and the syndrome of inappropriate antidiuretic hormone secretion (SIADH) (Cheng et al., 2011).

In a study conducted between 1985 and 1986 at the Oncology Service of the Veterans Administration Medical Center of Martinez, California, seven of seventy patients treated with cisplatin over 18 months developed RSWS and orthostatic hypotension. Symptoms started between 2 to 4 months of starting cisplatin. Patients were found to have a fractional excretion of sodium (FENa) exceeding 1% during volume depletion, which is in contrast to normal physiology where tubular reabsorption of sodium is increased substantially with hypovolemia achieving FENa <1%. Similar to our patient, hypovolemia occurred in the absence of extrarenal volume loss or inadequate oral intake. Volume depletion with persistent large urinary sodium loss was observed when intravenous fluid support was withdrawn rapidly, a finding consistent with renal salt wasting. The study concluded that RSWS is not uncommon and may occur in as many as 10% of patients receiving cisplatin (Hutchison et al., 1988).

The diagnosis of RSWS is difficult and is commonly misdiagnosed as SIADH as the conditions share similar laboratory values such as hypotonic hyponatremia and increased urine sodium. SIADH also occurs in many malignancies. Volume assessment is the most critical step in differentiating the conditions as patients with SIADH are euvolemic and those with RSWS are hypovolemic. Table 2 gives us a summary of how to differentiate between RSWS and SIADH. Management of RSWS and SIADH is quite different with the primary focus of treatment in RSWS being sodium chloride replacement while fluid restriction is most important in SIADH (Cheng et al., 2011).

**TABLE 2:** Differentiation of RSW and SIADH (Maesaka et al., 2009).

	RSW (in our patient)	SIADH
ECV	↓ (hypovolemic on examination)	N‐ ↑
UNa	N‐ ↑ (81 mEq/L)	N‐ ↑
Renin	_+/−_↑ (3.24 ng/mL/h)	_+/−_ ↓
Aldosterone	↑ (34 ng/dL)	_+/−_ ↓
Serum urate	↓↓ (7.8 mg/dL)	↓ ‐N
FEurate	↑↑ (not measured)	↑ ‐N
FEphosphate	_+/−_↑ (not measured)	N

Abbreviations: ECV, extracellular volume; RSW, renal salt wasting; SIADH, secretion of antidiuretic hormone; UNa, urinary sodium excretion.

RSWS likely involves multiple areas of the renal tubules and can affect the entire nephron including the glomeruli. This is not surprising when considering cisplatin renal metabolism and its characteristic as a heavy metal. Cisplatin is 90% protein bound but the remaining 10% is freely filtered through the glomeruli (Cheng et al., 2011; Hamdi et al., 2010). Cisplatin enters proximal tubular cells via basolateral organic cation transporter 2 and the copper transporter Ctr1, and distal tubular cells via Crt1 (Ciarimboli et al., 2005; Hamdi et al., 2010; Hutchison et al., 1988; Maryam Rahman & Friedman, 2009; Pabla et al., 2009; Soares et al., 2020). In addition, cisplatin is conjugated to glutathione and metabolized, generating molecules responsible for nephrotoxicity (Hamdi et al., 2010; Thurau & Boylan, 1976). Proximal tubular dysfunction will lead to impaired sodium and water reabsorption. The resultant increased sodium chloride delivery to the macula densa will in turn activate tubuloglomerular feedback which will lead to a decrease in GFR (Cornelison & Reed, 1993; Thurau & Boylan, 1976). Distal convoluted tubule dysfunction will further limit sodium chloride reabsorption, especially in light of the increased solute being delivered (Daugaard, 1990; Daugaard et al., 1988). Dysfunction in the loop of Henle can cause loss of the countercurrent gradient leading to a urinary concentrating defect and impairment in water reabsorption (Isnard‐Bagnis, 2003; Meyer & Madias, 1994). Cisplatin has also been reported to decrease the expression of the aquaporin water channels in the collecting ducts, leading to further impairment of water reabsorption (Kim et al., 2001). It was observed that cisplatin in rodents reduces the expression of aquaporin 2 and aquaporin 3. However, volume depletion in the setting of RSWS should increase vasopressin secretion and enhance urinary concentrating mechanisms.

Other mechanisms likely contribute to Cisplatin‐induced RSWS. In a study by Soares AG et al, it was proposed that Cisplatin decreases epithelial sodium channel (ENaC) activity, likely via a binding site on the channel (Soares et al., 2020). An alternative mechanism of ENaC dysfunction may be secondary to direct heavy metal toxicity induced by platinum (Soares et al., 2020).

Literature on the management of RSWS is sparse. Volume and sodium chloride remains the standard of care. Fludrocortisone is a synthetic adrenocortical steroid with mineralocorticoid properties that acts on the distal tubules of the kidney to enhance Na reabsorption (Maryam Rahman & Friedman, 2009). Fludrocortisone can be useful in RSWS by reducing natriuresis (Chua et al., 2018). In the article by Hamdi et al, all patients received intravenous normal saline with or without oral sodium supplements and experienced recovery of their acute kidney injury and electrolyte abnormalities within three days to three weeks. Fludrocortisone was used in three out of seventeen cases (Hamdi et al., 2010).

In an article by Hutchison et al, long‐term management of RSWS included the use of fludrocortisone (0.2–0.3 mg/day) or deoxycorticosterone in addition to oral salt replacement. The dose of fludrocortisone was 0.2–0.3 mg/day, and oral sodium intake ranged from 20 to 40 g sodium chloride per day. Patients were also instructed to monitor their urine volume and drink fluids based on the volume voided. Four out of the seven patients in the study passed away, but the remaining three continued to receive fludrocortisone and sodium chloride supplements 3, 7, and 24 months after diagnosis of RSWS (Hutchison et al., 1988). We also observed this in our patient, who continues to develop symptomatic hyponatremia and prerenal azotemia with attempts to taper his treatment. In summary, Cisplatin can impair both proximal and distal tubular Na absorption leading to RSWS. The diagnosis of RSWS should be considered in patients with hyponatremia and a high fractional excretion of sodium, particularly in patients with evidence of prerenal azotemia.

## AUTHOR CONTRIBUTIONS

Tissa Bijoy George involved in writing the manuscript and literature review. Kiran Kuriakose served as a member of care team and involved in writing manuscript. Anjana Chandrasekhara Pillai involved in writing the manuscript and literature review. Thomas Powell served as a member of care team, and involved in review and editing the manuscript—case presentation. Thomas R Kleyman involved in review and editing the manuscript discussion.

## FUNDING INFORMATION

This work was supported by a grant from the National Institutes of Health P30 DK079307.

## CONFLICT OF INTEREST STATEMENT

The authors have no conflict of interest to declare.

## ETHICS STATEMENT

A signed consent was obtained from the patient.
